# Distribution of vesicular glutamate transporters in the human brain

**DOI:** 10.3389/fnana.2015.00023

**Published:** 2015-03-05

**Authors:** Érika Vigneault, Odile Poirel, Mustapha Riad, Josée Prud'homme, Sylvie Dumas, Gustavo Turecki, Caroline Fasano, Naguib Mechawar, Salah El Mestikawy

**Affiliations:** ^1^Department of Psychiatry, Douglas Mental Health University Institute, McGill UniversityMontreal, QC, Canada; ^2^Institut National de la Santé et de la Recherche Médicale, UMR-S 1130, Neuroscience Paris SeineParis, France; ^3^Centre National de la Recherche Scientifique UMR 8246, Neuroscience Paris SeineParis, France; ^4^Sorbonne University, Université Pierre et Marie Curie Paris 06, UM119, Neuroscience Paris SeineParis, France; ^5^OramacellParis, France

**Keywords:** *Homo sapiens*, brain, neurotransmission, glutamate, VGLUTs, anatomy

## Abstract

Glutamate is the major excitatory transmitter in the brain. Vesicular glutamate transporters (VGLUT1-3) are responsible for uploading glutamate into synaptic vesicles. VGLUT1 and VGLUT2 are considered as specific markers of canonical glutamatergic neurons, while VGLUT3 is found in neurons previously shown to use other neurotransmitters than glutamate. Although there exists a rich literature on the localization of these glutamatergic markers in the rodent brain, little is currently known about the distribution of VGLUT1-3 in the human brain. In the present study, using subtype specific probes and antisera, we examined the localization of the three vesicular glutamate transporters in the human brain by *in situ* hybridization, immunoautoradiography and immunohistochemistry. We found that the VGLUT1 transcript was highly expressed in the cerebral cortex, hippocampus and cerebellum, whereas VGLUT2 mRNA was mainly found in the thalamus and brainstem. VGLUT3 mRNA was localized in scarce neurons within the cerebral cortex, hippocampus, striatum and raphe nuclei. Following immunoautoradiographic labeling, intense VGLUT1- and VGLUT2-immunoreactivities were observed in all regions investigated (cerebral cortex, hippocampus, caudate-putamen, cerebellum, thalamus, amygdala, substantia nigra, raphe) while VGLUT3 was absent from the thalamus and cerebellum. This extensive mapping of VGLUT1-3 in human brain reveals distributions that correspond for the most part to those previously described in rodent brains.

## Introduction

Glutamate, the major excitatory neurotransmitter in the brain, has been implicated in many neurological and psychiatric disorders (Fonnum, 1984). This amino acid is present in all cell types and participates in numerous cellular functions, from protein biosynthesis to various aspects of metabolism. As a *bona fide* neurotransmitter, glutamate must first be uploaded into synaptic vesicles within presynaptic terminals before undergoing regulated release at the synaptic cleft (Fonnum et al., 1998; Gasnier, 2000). Proton-dependent carriers, named vesicular glutamate transporters (VGLUTs) 1–3, are responsible for the vesicular accumulation of glutamate (for review see El Mestikawy et al., 2011; Hnasko and Edwards, 2012). These key actors of excitatory neurotransmission were first identified and characterized in rodents and humans (Ni et al., 1994; Bellocchio et al., 1998, 2000; Aihara et al., 2000; Takamori et al., 2000, 2001, 2002; Fremeau et al., 2001, 2002; Gras et al., 2002; Schafer et al., 2002; Varoqui et al., 2002). Takamori and colleagues established more than a decade ago that the expression of a vesicular glutamate transporter in neurons was sufficient to confer a “glutamatergic phenotype” (Takamori et al., 2000, 2001). Moreover, alteration of VGLUT levels has been shown to modify the glutamate content of synaptic vesicles and hence the strength of excitatory synaptic transmission (Daniels et al., 2004). VGLUTs are thus key anatomical and functional markers of glutamatergic transmission.

With the advent of genetically engineered mice, specific features and functions of the 3 VGLUTs have recently been identified (for review see Wallen-Mackenzie et al., 2010). VGLUT1-knockout mice (VGLUT1-KO) die after weaning (Fremeau et al., 2004b; Wojcik et al., 2004) while VGLUT2-KO die immediately after birth from respiratory insufficiency (Moechars et al., 2006; Wallen-Mackenzie et al., 2006). These findings have clearly established that VGLUT1 and VGLUT2 are engaged in the regulation of vital functions. In contrast, VGLUT3-KO mice have a normal life expectancy despite being deaf and developing anxiety and discrete basal locomotor phenotypes (Ruel et al., 2008; Seal et al., 2008; Amilhon et al., 2010). Recent studies using constitutive or conditional knock-out mouse models have further established that the 3 VGLUTs are involved in cognition, mood regulation, locomotor activity, reward, and pain (for reviews, see Seal and Edwards, 2006; Benarroch, 2010; Wallen-Mackenzie et al., 2010; El Mestikawy et al., 2011).

The anatomical distribution of the 3 VGLUTs has been thoroughly characterized in the mature rodent brain (for review see El Mestikawy et al., 2011). To briefly summarize, a large population of cortical excitatory neurons expresses VGLUT1, whereas subcortical glutamatergic neurons express VGLUT2 (Aihara et al., 2000; Herzog et al., 2001). In addition, VGLUT2 is also found in some cortical neurons (predominantly layer IV) while VGLUT1 is expressed in some thalamic nuclei (Fremeau et al., 2001). Together, VGLUT1- and VGLUT2-expressing neurons represent the bulk of “canonical” glutamatergic neurons in the brain. VGLUT1 represents the major subtype and accounts for approximately 80% of total vesicular transport of glutamate in the brain (Fremeau et al., 2004b). Combined, VGLUT1- and VGLUT2-immunoreactive (-IR) neurons extend nearly ubiquitous projections throughout the brain. In contrast, the distribution of VGLUT3-immunoreactivity is much more discrete, as it is found in discrete population of neurons using other transmitters than glutamate (Fremeau et al., 2002; Gras et al., 2002; Schafer et al., 2002; Takamori et al., 2002). VGLUT3 is expressed by cholinergic interneurons in the dorsal and ventral striatum, subpopulations of GABAergic basket cells in the cortex and hippocampus, and serotoninergic neurons (for review, see El Mestikawy et al., 2011). However, It should be kept in mind that VGLUT1 and VGLUT2 as well are expressed by “non-glutamatergic” neurons (Boulland et al., 2009; Fattorini et al., 2009; Zander et al., 2010; Ren et al., 2011).

The almost non-overlapping distribution of the 3 VGLUTs delineates three complementary glutamatergic systems. VGLUT1 and VGLUT2 play major neurophysiological roles in virtually all major neuronal circuits, while VGLUT3 is involved in more subtle modulation of local transmission (for review see El Mestikawy et al., 2011).

The widespread distributions of VGLUT1-3 in rodent CNS suggest their involvement in the regulation of sensori-motor, cognitive and mood processes. Hence, it can be surmised that alterations in the expression of these proteins may underlie significant aspects of human neurolopathologies. Indeed, recent postmortem studies have highlighted alterations in VGLUT1 and VGLUT2 expression in mood disorders and psychosis (Oni-Orisan et al., 2008; Uezato et al., 2009; Eastwood and Harrison, 2010) as well as in neurological disorders such as Parkinson disease, Alzheimer disease and epilepsy (Kirvell et al., 2006; Kashani et al., 2007, 2008; Van Der Hel et al., 2009). These studies measured VGLUT1-3 mRNA or VGLUT1 and VGLUT2 protein in cortical or subcortical areas. Unfortunately, the significance of such results is limited by the fact that our current anatomical knowledge of VGLUT-defined glutamatergic subsystems in the human brain is partial at best. The aim of the present study was thus to characterize the distributions of VGLUT1-3 transcripts and proteins in major regions of the human brain.

## Materials and methods

### Human brain samples

This study was approved by the Douglas Institute Research Ethics Board, and written informed consent from next-of-kin was obtained in each case. Postmortem brain samples from six caucasian individuals (Table 1) were provided by the Douglas-Bell Canada Brain Bank (www.douglasbrainbank.ca). Three of the individuals died from accidents or natural causes, with one having been diagnosed with major depression and two who had no history of neurological nor psychiatric illness. The two other individuals committed suicide during a depressive episode (Table 1). After extraction, brains were immediately placed at 4°C, rapidly sent to the Douglas-Bell Canada Brain Bank where they were sliced into thick sections, rapidly frozen and stored at −80°C until further use. The postmortem refrigeration delay, i.e., the time between death and storage of the body at 4°C at the morgue, was of 15.5 ± 8.6 h (±SEM) on average. Frozen samples from the following brain regions were dissected from the left hemisphere by expert brain bank staff, cut on a cryostat in 10 μm-thick serial sections, and processed for *in situ* hybridization or immunoautoradiography (IAR), as detailed below: cerebral cortex (Brodmann areas [BAs] 4 and 9), hippocampus, striatum, amygdala, cerebellum, substantia nigra, thalamus, raphe.

**Table 1:** **Subject information**.

**Subject**	**Gender**	**Age (years)**	**Diagnosis**	**Cause of death**	**RD (h)**
135	Male	18	Nil	Natural death cardiovascular^*^	2.0
138	Female	66	Nil	Car accident^*^	57.6
146	Male	26	SCZ	Suicide^*^	3.9
152	Female	49	MDD	Suicide^*^	14.7
155	Male	31	MDD	Natural death cause unknown^*^	9.2
513	Male	72	Nil	Heart attack	5.0

* *As recorded by the coroner. MDD, major depressive disorder; Nil, no psychiatric nor neurological disorders; RD, refrigeration delay; SCZ, schizophrenia*.

### *In situ* hybridization

*In situ* hybridization was performed as recommended by Oramacell (Paris, France) and as previously reported (Gras et al., 2008). In brief, sense or antisense oligonucleotides specific for human VGLUT1, VGLUT2, or VGLUT3, the dopamine (DAT) or serotonin (SERT) transporters (**Table 3**) (generated by Oramacell) were labeled with [^35^S]-dATP (GE Healthcare) using terminal deoxynucleotidyl transferase (GE Healthcare) to a specific activity of 1–3 × 10^8^ dpm/mg. On the day of the experiment, fresh frozen section (10 μm) were fixed in 3.7% formaldehyde in phosphate-buffered saline (PBS), washed with PBS, rinsed with water, dehydrated in 50 and 70% ethanol, and air-dried. Sections were then covered with 140 μl of hybridization medium (Oramacell, Paris, France) containing 1.4 μl of the labeled oligonucleotide. Slides were incubated 16 h at 42°C, washed 2 × 15 min in saline sodium citrate buffer (SSC 1X) and dithiothreitol (DTT, 10 mM) at 53°C, 2 × 15 min in SSC 0.5X, dehydrated in ethanol, air-dried and exposed to a BAS-SR Fujifilm Imaging Plate for 7–10 days for VGLUT1 and VGLUT2, 21 days for VGLUT3, 14 days for the dopamine transporter (DAT) and for 4 days for the serotonin transporter (SERT) mRNA. The plates were scanned with a Fujifilm BioImaging Analyser BAS-5000. Each marker was investigated with a combination of 4–5 antisense or sense oligonucleotides (Table 2). In preliminary experiments, each antisense or sense oligonucleotide was incubated individually or as pool of sense or antisense nucleotides for a given marker. A labeling was considered specific when: (i) the distribution observed for each individual oligonuleotide were similar as the one obtained with combined 4 oligonucleotides and (ii) no labeling was obtained with corresponding sense nucleotides.

**Table 2:** **List of oligonucleotides used for *in situ* hybridization**.

**HUMAN VGLUT1**
**Antisense oligonucleotides**
HV1-AS1: GGGACTCGTAGGAGACGAGCAGCCAGAACAGGTAC
HV1-AS2: GAGAGCACGACCCGCTAGCTTCCGAAACTCCTCCT
HV1-AS3: GAGCAGGGTTCCTTGACACTGTCACTCAGGCCAG
HV1-AS4: CCCCGTAGAAGATGACACCTCCATAGTGCACCAGGG
**Sense oligonucleotides**
HV1-S1: GTACCTGTTCTGGCTGCTCGTCTCCTACGAGTCCC
HV1-S2: AGGAGGAGTTTCGGAAGCTAGCGGGTCGTGCTCTC
HV1-S3: CTGGCCTGAGTGACAGTGTCAAGGAACCCTGCTC
HV1-S4: CCCTGGTGCACTATGGAGGTGTCATCTTCTACGGGG
**HUMAN VGLUT2**
**Antisense oligonucleotides**
HV2-AS1: GCTTCTTCTCCAGACCCCTGTAGATCTGGCCGAG
HV2-AS2: GAAGGGGAGTATCCGGTGGCAAAGAGCGCAAGCAG
HV2-AS3: CCCCAAAAGAAGGAACCGTGGATCATCCCCACGG
HV2-AS4: CTTTCTCCTTGATGACCTTGCCCCCGCGGTGGAT
**Sense oligonucleotides**
HV2-S1: CTCGGCCAGATCTACAGGGTGCTGGAGAAGAAGC
HV2-S2: CTGCTTGCGCTCTTTGCCACCGGATACTCCCCTTC
HV2-S3: CCGTGGGGATGATCCACGGTTCCTTCTTTTGGGG
HV2-S4: ATCCACCGCGGGGGCAAGGTCATCAAGGAGAAAG
**HUMAN VGLUT3**
**Antisense oligonucleotides**
HV3-AS1: CGGCTTCTCTCCAAAGGTGGTGCCCACTTA
HV3-AS2: AGCCAACCACCAGGAGTAAGGTTGCCTCCATGCC
HV3-AS3: CCCCTCCCAATATTTGGACCTCTGGCAAGCTGGG
HV3-AS4: GGACCATCCAATGTACTGCACCAACACCCCAGCC
**Sense oligonucleotides**
HV3-S1: GGAGTAAGTGGGCACCACCTTTGAGAGAAGCCG
HV3-S2: GGCATGGAGGCAACCTTACTCCTGGTGGTTGGCT
HV3-S3: CCCAGCTTGCCAGAGGTCCAAATATTGGGAGGGG
HV3-S4: GGCTGGGGTGTTGGTGCAGTACATTGGATGGTCC
**HUMAN DAT**
**Antisense oligonucleotides**
DAT1AS1: GACTTCCTGGGGTCTTCGTCTCTGCTCCCTCTAC
DATAS2: GTAGGCCAGTTTCTCTCGAAAGGACCCAGGCAGG
DATAS3: GGTATGCTCTGATGCCGTCTATGGCTCCAGGGAG
DATAS4: GCCTGAGTGGCAGTAGCCTGAGCTGGTTTCAAGG
DATAS5: GTTGGCCCAGTCGGGGAAGATGTAGGCTCCGTAGT
**Sense oligonucleotides**
DAT1S1: GTAGAGGGAGCAGAGACGAAGACCCCAGGAAGTC
DATS2: CCTGCCTGGGTCCTTTCGAGAGAAACTGGCCTAC
DATS3: CTCCCTGGAGCCATAGACGGCATCAGAGCATACC
DATS4: CCTTGAAACCAGCTCAGGCTACTGCCACTCAGGC
DATS5: ACTACGGAGCCTACATCTTCCCCGACTGGGCCAAC
**HUMAN SERT**
**Antisense oligonucleotides**
humSERT-AS1: AGCCACTAGGGTGGTGGTGGTCGCTGGGATAGAGT
humSERT-AS2: CCTCCGAGCTCTCTATCGTCGGGATTGACACGTC
humSERT-AS3: CAGGACCCCAAAGCCCGGACCAAGAGAGAAGAAG
humSERT-AS4: GAACAGGAGAAACAGAGGGCTGATGGCCACCCAG
**Sense oligonucleotides**
humSERT-S1: ACTCTATCCCAGCGACCACCACCACCCTAGTGGCT
humSERT-S2: GACGTGTCAATCCCGACGATAGAGAGCTCGGAGG
humSERT-S3: CTTCTTCTCTCTTGGTCCGGGCTTTGGGGTCCTG
humSERT-S4: CTGGGTGGCCATCAGCCCTCTGTTTCTCCTGTTC

### Immunoautoradiography

**Figure 1:**
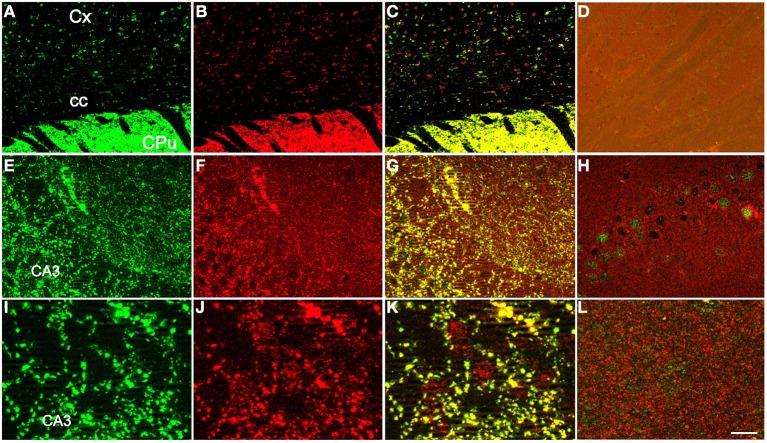
**Specificity of the newly developed anti-human VGLUT3 antiserum**. The distribution of VGLUT3-positive immunolabeling was compared between the purified serum newly produced in rabbit against a human peptide **(A,E,I)** and a previously validated rodent anti-VGLUT3 in guinea pig **(B,F,J)**, (Gras et al., 2008; Amilhon et al., 2010; Miot et al., 2012). Immunofluorescence experiments were conducted as previously described (Gras et al., 2008; Amilhon et al., 2010; Miot et al., 2012) on sections taken at the level of the striatum **(A–D)** or hippocampus **(E–L)** of wild-type **(A–C,E–G,I–K)** or VGLUT3-null mice **(D,H,L)**. The newly developed anti-human VGLUT3 was visualized with red fluorescence (anti rabbit IgG-Alexa fluor 555) and the rodent isoform with green fluorescence (anti-guinea pig IgG-Alexa fluor 488). The merged channels are shown in **(C)** for the caudate-putamen and in **(G,K)** for the hippocampus. Scale bar (in **L**): 100 μm in **(A–D)**, 30 μm in **(E–H)**, and 10 μm in **(I–L)**. Abbreviations: CA3, CA3 field of the pyramidal layer of the hippocampus; cc, corpus callosum; CPu, caudate putamen; Cx, cerebral cortex.

To visualize proteins, immunoautoradiography (IAR) was performed as previously described (Kashani et al., 2007, 2008). Selective antibodies against VGLUT1 and VGLUT2 were obtained by immunization of rabbits with the corresponding peptides, as described previously (Herzog et al., 2001; Kashani et al., 2007, 2008). To detect VGLUT3 in human samples an antiserum was developed by immunizing rabbits with the N-CEEIELNHESFASPKKKM-C peptide fused to the Keyhole limpet haemocyanin (AgroBio, Villeny, France, http://www.agro-bio.fr/). The anti-VGLUT3 antiserum was affinity purified on an Affigel-10 (Biorad, Richmond, CA) matrix coupled to the N-CEEIELNHESFASPKKKM-C peptide as described (Kashani et al., 2007). The specificity of the newly developed serum was assessed by comparing the distribution of VGLUT3-positive immunolabeling obtained with the serum newly produced against a human peptide (in rabbit) and a previously validated rodent anti-VGLUT3 (in guinea pig, Gras et al., 2008; Amilhon et al., 2010; Miot et al., 2012). Immunofluorescence experiments were conducted as previously described on wildtype or VGLUT3-nul mouse brain (Figure 1; Gras et al., 2008; Amilhon et al., 2010; Miot et al., 2012). As shown in Figures 1A–C,E–G,I–K the immunodistribution of the new anti-human VGLUT3 antiserum overlapped with the known labeling of VGLUT3 in both mouse hippocampus and striatum. Furthermore, no labeling was observed with the new anti-human VGLUT3 antiserum on sections of mouse brain that no longer expressed VGLUT3 (Figures 1D,H,L; Gras et al., 2008). This experiment demonstrated that the new serum binds to VGLUT3 and rules out cross-reactivity with VGLUT1 and VGLUT2 or any other brain protein.

Anti-VGLUT1 was used at 1:2000; anti-VGLUT2 affinity purified antiserum at 1:4000 (Kashani et al., 2007, 2008) and the affinity purified anti-VGLUT3 at 1:4000.

On the day of the experiment, fresh frozen sections (10 μm) were fixed in 4% formaldehyde in PBS, washed with PBS, and preincubated in PBS containing 3% bovine serum albumin, 1% normal goat serum and 1 mM NaI (buffer A) for 1 h. Sections were incubated with buffer A supplemented with polyclonal rabbit anti-VGLUT1-3 antisera overnight at 4°C, followed by anti-rabbit [^125^I]-IgG (GE Healthcare lifesciences, 100 mCi/ml) for 2 h. Sections were exposed to X-ray films (Biomax MR, Kodak, France) for 24 h, and images were digitized using a PowerLook 100 Umax scanner and analyzed with Multi Gauge Software.

### Immunohistochemistry

Immunohistochemistry detection of VGLUT1-3 on human brain sections was performed as previously described with minor modifications. Paraffin embedded sections (5 μm) of the cerebellum were obtained from formalin-fixed brains as already described (Torres-Platas et al., 2014). VGLUT1-3 immunostaining was performed with the anti-VGLUT1 antiserum (1:500 dilution) as described previously (Kashani et al., 2008) and then processed with biotinylated goat anti-rabbit IgG (1:500 dilution), streptavidine (1:1000 dilution) and 3′3-diaminobenzidine reagents (Jackson Immunoresearch, Brulington Ontario, Canada). Images were obtained with a Zeiss Axioscop microscope.

## Results

### General considerations

The overall regional distributions of VGLUT1-3 mRNA and proteins were highly similar between subjects (Table 3), and only slight inter-individual variability in the intensity of signals was noticed, irrespective of gender, age, presence/absence of psychiatric disorder, or cause of death.

**Table 3:** **Distribution of VGLUT1-3 transcripts and proteins in various area of the human brain**.

**Transporter**	**VGLUT1**		**VGLUT2**		**VGLUT3**		**Subject**
**Region**	**mRNA**	**Protein**	**mRNA**	**Protein**	**mRNA**	**Protein**	
Cerebral cortex	+++	+++	+	++	+	++	146
Hippocampus	+++	+++	N.D.	++	+	+++	146 (**Figure 3**)
							155 (**Figure 4**)
Amygdala	+ (LA)	+++	N.D.	++	N.D.	++	135
Basal Ganglia	N.D.	+++	N.D.	+++	+	+++	152
							155
Habenula	N.D.	+	+++	+++	N.D.	+++	146
SNC	N.D.	+	N.D.	+++	N.D.	+++	
Thalamus	N.D.	++	+++	++	N.D.	N.D.	138
Raphe	++ (Pons)	++	+++ (Pons)	+++	++	+++	146
Cerebellum	+++ (Gcl)	+++	N.D.	+++	N.D.	N.D.	146

*LA, lateral amygdala; Gcl, granular cell layer of the cerebellum; N.D., not detected*.

### Regional VGLUT1-3 mRNA and protein distributions

#### Cerebral cortex (BA4, BA9)

**Figure 2:**
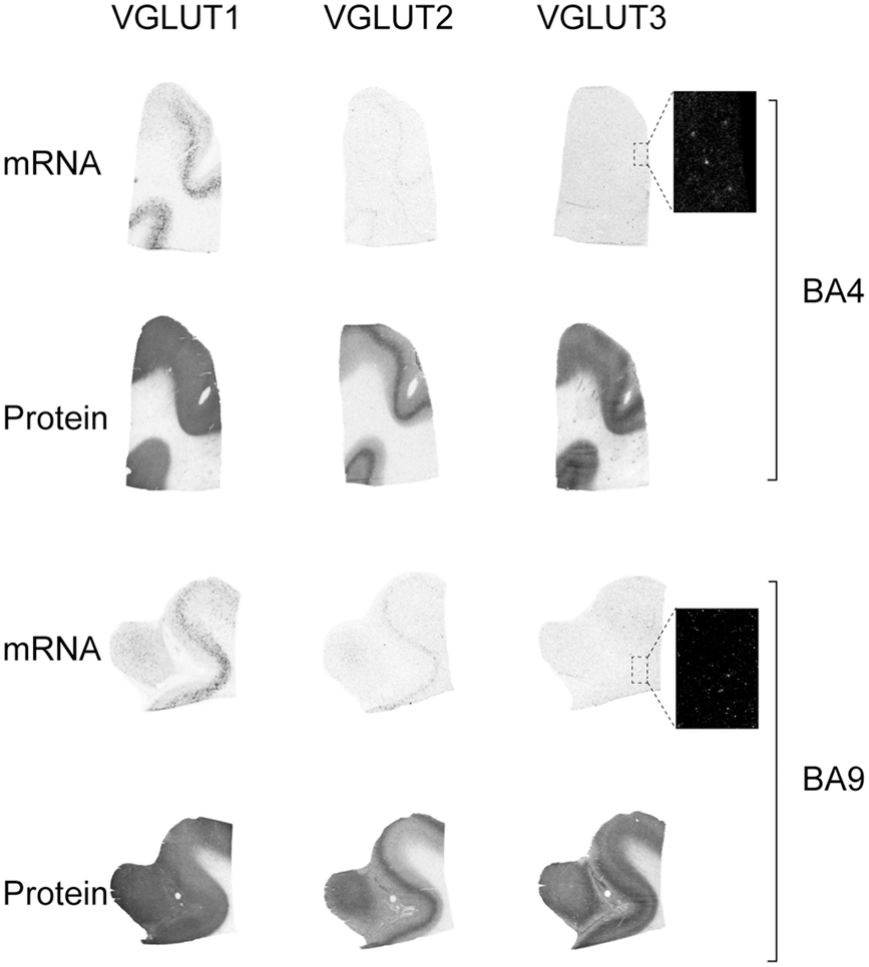
**Distribution of VGLUT1-3 mRNA and protein in cerebral cortical Brodmann areas (BA)4 and BA9**. Boxed regions are enlarged and shown in negative.

The expression patterns of each of the three VGLUTs were conserved in both motor (BA4) and prefrontal associative cortex (BA9) (Figure 2). VGLUT1 mRNA was absent in upper layers but abundant in layers V–VI, with varying intensity levels. Sparse labeling was also occasionally observed in the underlying white matter. VGLUT1 protein detected by IAR was strongly and homogeneously distributed throughout the cortical thickness, but absent in white matter.

VGLUT2 mRNA was restricted to a band of cells spanning lower layer V. This distribution was mirrored by the presence of a more abundant cortical VGLUT2-immunoreactive material in layer V. The other cortical layers were uniformly immunoreactive but less intensely than layer V. In contrast to VGLUT1 and VGLUT2, VGLUT3 mRNA was only observed in scarce cells across the gray matter. VGLUT3 protein, however, was observed throughout the cortex with particular abundance in upper and lower layers, above and below of a band of lower intensity in mid-cortex.

#### Hippocampus

**Figure 3:**
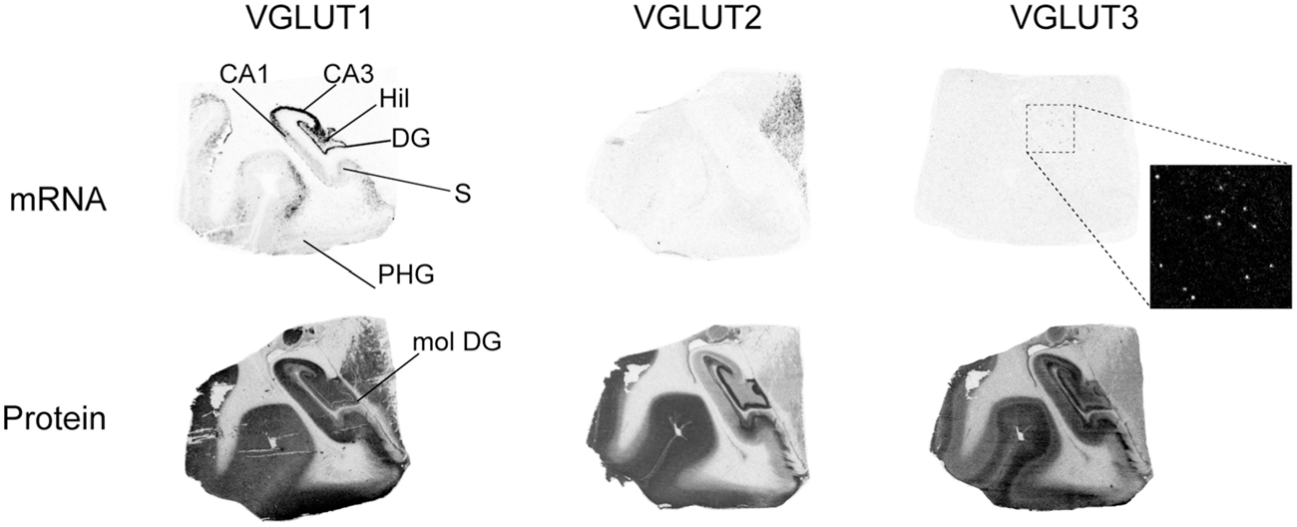
**Distribution of VGLUT1-3 mRNA and protein in the hippocampal formation**. Boxed regions are enlarged and shown in negative. Abbreviations: CA1–3, fields of the pyramidal layer of the hippocampus; DG, dentate gyrus; Hil, hilus of the dentate gyrus; mol DG, molecular layer of the dentate gyrus; PHG, parahippocampal gyrus; S, subiculum.

In the hippocampal formation, VGLUT1 mRNA was detected in the pyramidal layer of Cornu Ammonis (CA1-3) fields, in the granule cell layer of the dentate gyrus (DG), as well as in sparse cells across the hilus (Figure 3). Weak levels of transcripts were observed in the presubiculum. VGLUT1 protein was much more widely distributed, with strong immunoreactivity observed throughout gray matter, except in the granule cell layer of the DG and in white matter tract of the stratum lacunosam molecular above the molecular layer of the DG.

Although VGLUT2 mRNA was not detected in the hippocampus, the protein was very strongly expressed in the molecular layer of the DG, as well as in the mossy fiber pathway and in the subiculum.

VGLUT3 mRNA was only expressed in scattered cells throughout the hippocampal formation, except within the CA pyramidal cell layer and granule cell layer of the DG. The pattern of VGLUT3-IR was radically different, with more widespread distribution in gray matter displaying particularly strong signal in the outer DG granule cell layer, hilus, and mossy fiber pathway.

**Figure 4:**
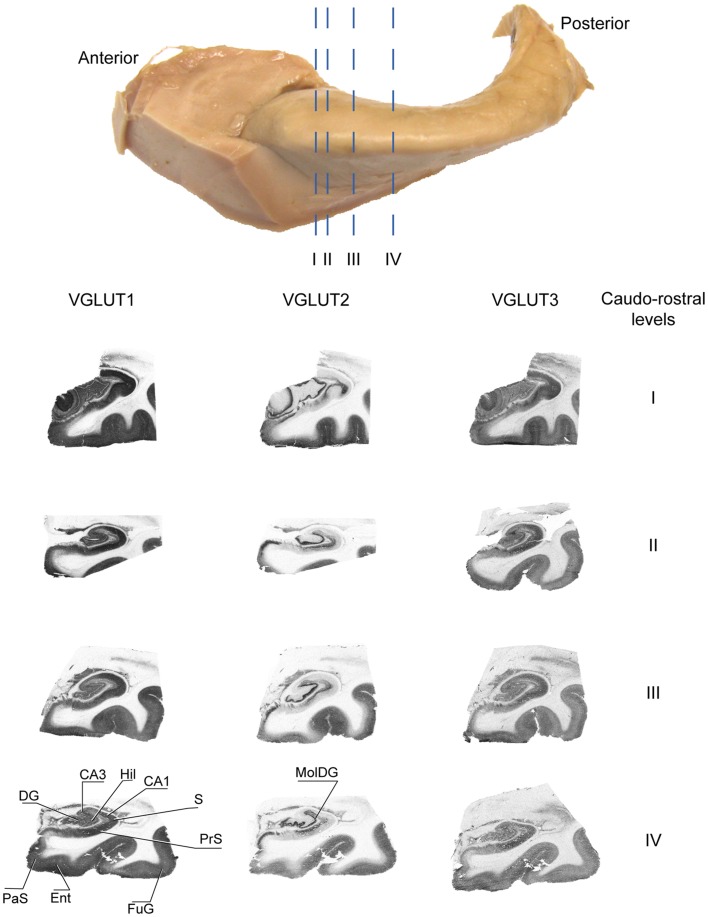
**Distribution of VGLUT1-3 proteins at various levels of the antero-posterior axis of the hippocampus**. Sections immunolabeled for VGLUT1, VGLUT2, and VGLUT3 were obtained at anatomical levels corresponding approximately to etched lines I-IV. Abbreviations: CA1–3, fields of the pyramidal layer of the hippocampus; DG, dentate gyrus; Ent, entorhinal cortex; FuG, fusiform gyrus; Hil, hilus; MolDG, molecular layer of the dentate gyrus; PaS, parasubiculum; PRC, perirhinal cortex; PrS, presubiculum; S, subiculum.

These patterns of expression were observed at various levels of the anteroposterior axis of the hippocampus (Figure 4).

#### Amygdala

**Figure 5:**
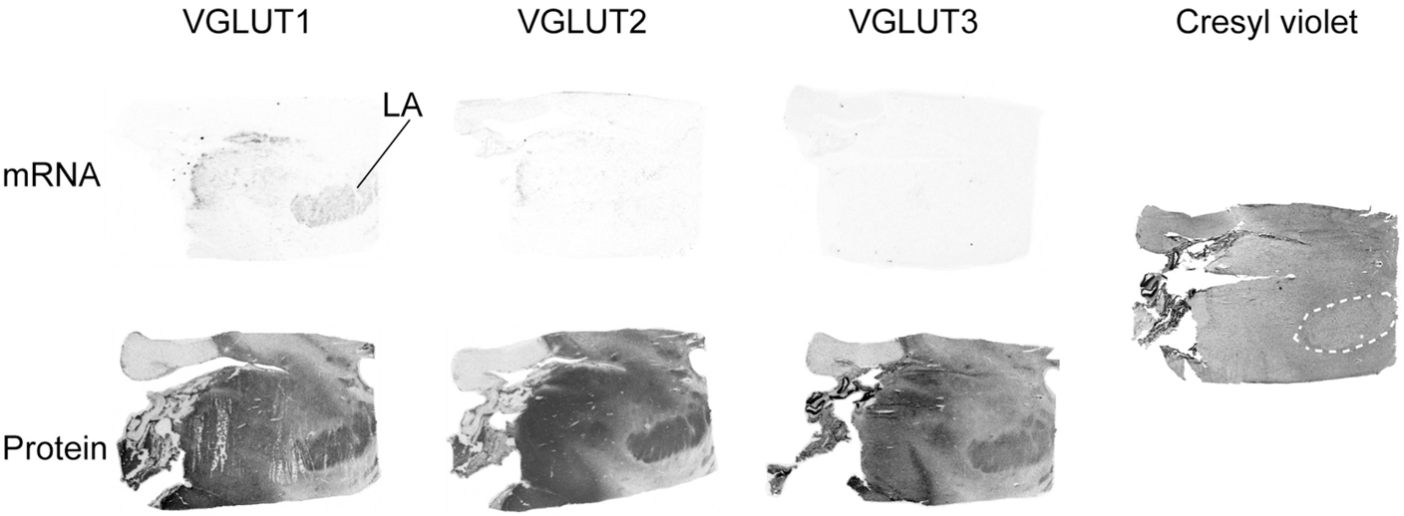
**Distribution of VGLUT1-3 mRNA and protein in the amygdala**. In addition to the in HIS and immunoautoradiographic labeling, the histological staining of an adjacent section is shown on the left. Abbreviation: LA, lateral amygdala.

Of the three transporters examined, VGLUT1 was the only one to display mRNA expression in the amygdala, with very faint signal being mostly restricted to the lateral amygdala. In contrast, all three VGLUT proteins were labeled in the amygdala. Immunoreactivities were prominent in the basolateral complex, although VGLUT3-IR was weaker than VGLUT1- and VGLUT2-IR (Figure 5).

#### Basal ganglia

**Figure 6:**
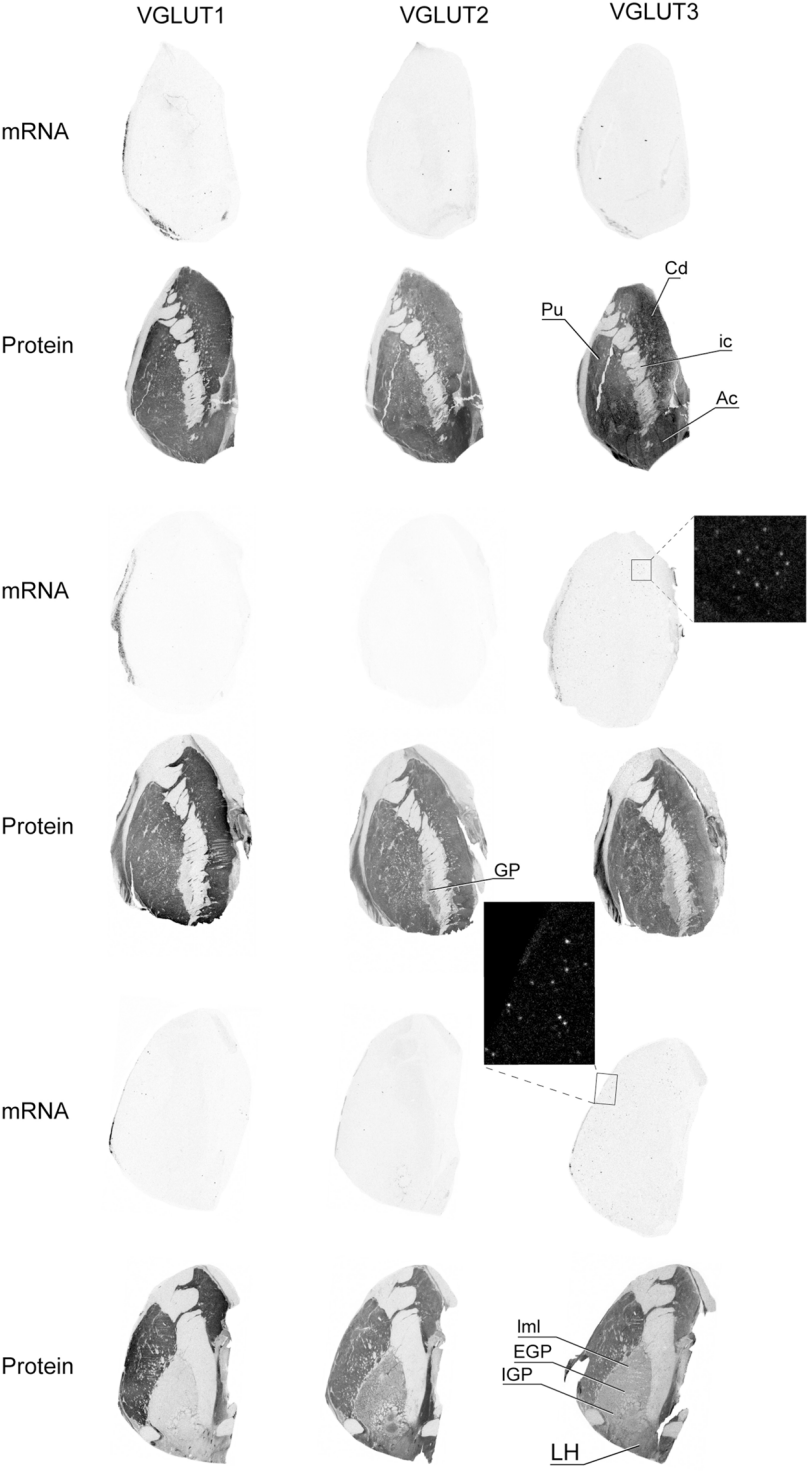
**Distribution of VGLUT1-3 mRNA and protein in the caudate-putamen region**. Boxed regions are enlarged and shown in negative. Abbreviations: Ac, nucleus accumbens; Cd, caudate nucleus; EGP, external globus pallidus; GP, globus pallidus; ic, internal capsule; IGP, internal globus pallidus; LH, lateral hypothalamus; lml, lateral medullary lamina of globus pallidus; Pu, putamen.

VGLUT1 and VGLUT2 transcripts were absent from basal ganglia whereas VGLUT3 mRNA was detected in sparse cells distributed in the caudate the putamen and the nucleus accumbens (Figure 6). Immunoreactivity was abundant for all three VGLUT proteins in the caudate, putamen, and nucleus accumbens. In the globus pallidus, VGLUT1-IR was absent, whereas VGLUT2 and VGLUT3 were weakly expressed in both external and internal segments. These patterns of expression were observed throughout the anteroposterior axis.

**Figure 7:**
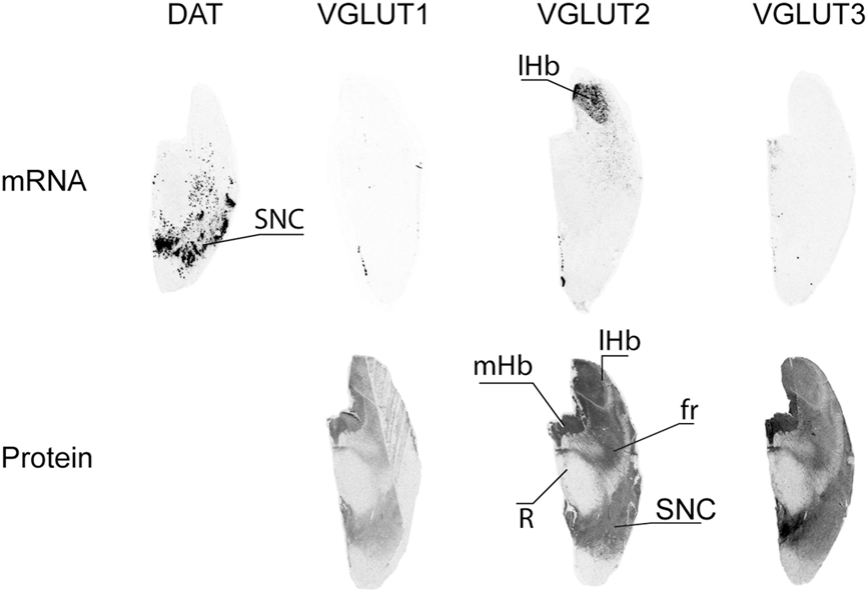
**Distribution of VGLUT1-3 mRNA and protein in substantia nigra**. Substantia nigra *pars compacta* (SNC) was identified by detection of the transcript coding for the dopamine transporter (DAT). Abbreviations: fr, fasciculus retroflexus; lHb, lateral habenula; mHb, medial habenula; R, red nucleus.

Dopaminergic cells from the substantia nigra *pars compacta* (SNC) were readily observed following *in situ* hybridization performed with antisense oligonucleotides targeting DAT (Figure 7). None of the 3 VGLUTs was found to display mRNA expression in adjacent sections of the substantia nigra. However, all three transporters presented diffuse IR with transporter-specific intensities. Thus, VGLUT1 and VGLUT3 displayed weak and strong immunoreactivities, respectively, whereas VGLUT2 presented intermediate labeling. The only labeling heterogeneity observed within this nucleus was for VGLUT3-IR, which seemed stronger in the SN pars medialis (Figure 7).

#### Habenula

The habenular complex displayed strong content of VGLUT2 mRNA transcripts, and absence of VGLUT1 and VGLUT3 mRNAs. All three VGLUT proteins were labeled in the habenula. VGLUT2-IR was very strong in both the lateral and medial habenula, VGLUT3-IR was high and moderate in the medial and lateral portions of the habenula, respectively, and faint VGLUT3-IR was restricted to the medial part of the complex (Figure 7). VGLUT2 mRNA was also observed in sparse cells located ventrally to the habenula.

#### Thalamus

**Figure 8:**
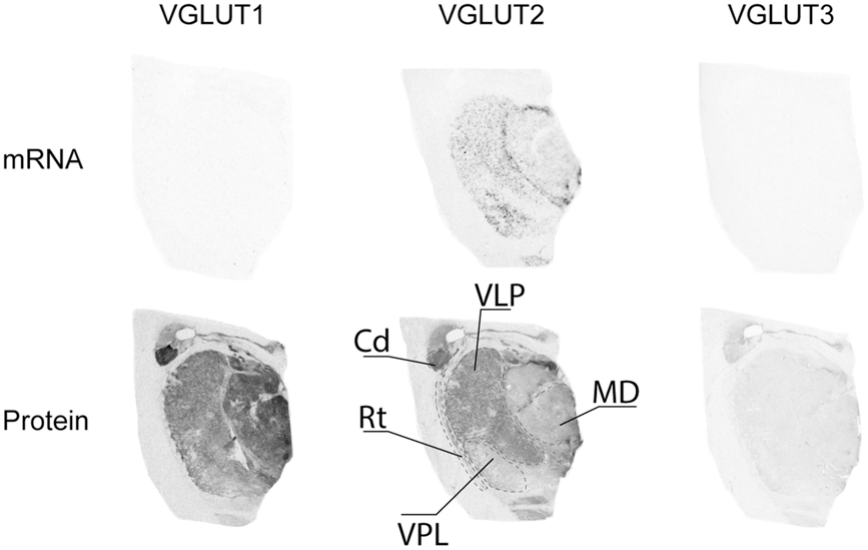
**Distribution of VGLUT1-3 mRNA and protein in the thalamus**. Abbreviations: Cd, caudate nucleus; MD, mediodorsal thalamic nucleus; Rt, reticular thalamic nucleus; VLP, ventral lateral posterior thalamic nucleus; VPL, ventral posterior thalamic nucleus.

Of the three VGLUTs, only VGLUT2 mRNA was found to be expressed in the thalamus (Figure 8). It was mainly present in scattered cells in the ventral lateral posterior (VLP) nucleus as well as the in the ventral posterior lateral (VPL) nucleus. Moderate VGLUT2-IR was moderate in the VLP, but extended beyond the extent of transcript distribution. VGLUT2-IR was also observed, albeit more weakly, in the mediodorsal thalamic nucleus. Despite the absence of transcript in this region, VGLUT1 protein was clearly expressed in all thalamic nuclei in the sampled region, with the exception of the reticular nucleus. VGLUT1-IR was strongest in the mediodorsal nucleus. Very faint and diffuse VGLUT3-IR was detected in the thalamus, with no particular pattern of expression.

#### Raphe

**Figure 9:**
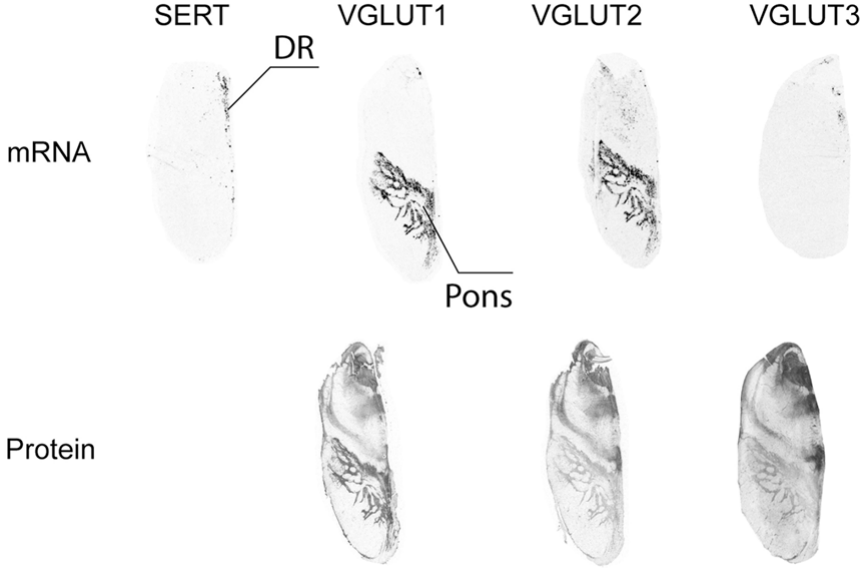
**Distribution of VGLUT1-3 mRNA and protein in the raphe**. Serotoninergic cells in the dorsal raphe nucleus (DR) were identified by detection of the transcript coding for the serotonin transporter (SERT).

Serotonergic neurons were observed by SERT ISH in the raphe. This area (raphe dorsalis) was partially labeled by VGLUT3 ISH, but did not display VGLUT1 nor VGLUT2 mRNA (Figure 9). This indicates that VGLUT3 is expressed only in a subset of 5HT neurons, as previously described in rodents (Kiyasova et al., 2011). In the same sections, the pons was found to be strongly labeled by VGLUT1 and VGLUT2 ISH, whereas VGLUT3 ISH revealed a complete absence of transcript. In contrast, all three VGLUT proteins were distributed in the pons in a similar and overlapping fashion, but with varying degrees of intensity. Thus, VGLUT1 immunoreactivity was the strongest, followed by VGLUT3- and VGLUT2-immunoreactivity (Figure 9).

#### Cerebellum

**Figure 10:**
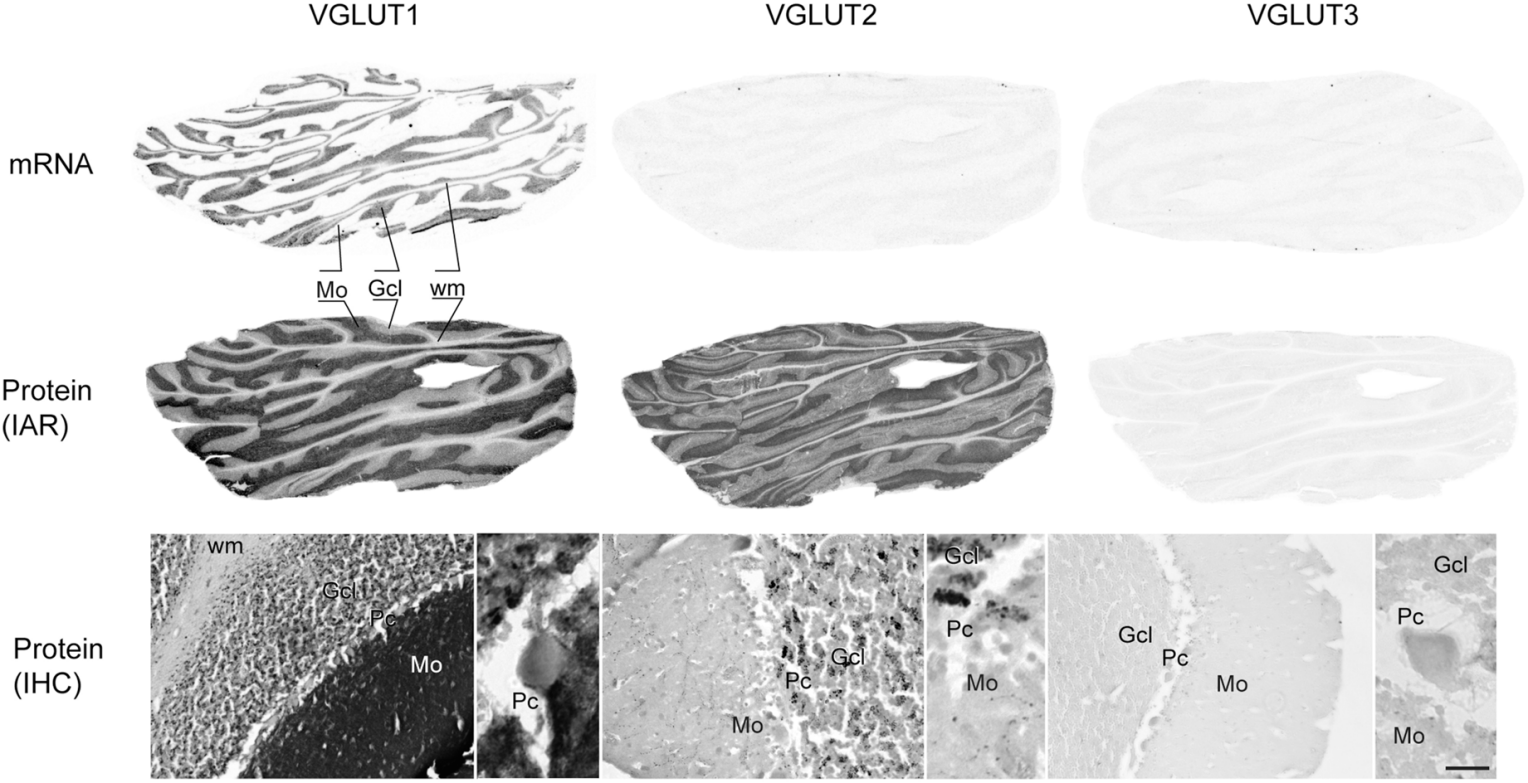
**Distribution of VGLUT1-3 mRNA and protein (immunoautoradiography [IAR] and immunohistochemistry [IHC]) in the cerebellum**. Abbreviations: GcL, granular cell layer of the cerebellum; Mo, molecular layer of the cerebellum; Pc, Purkinje cell layer; Pk, Purkinje cell; wm, white mater.

In the cerebellum, VGLUT1 mRNA was detected only in the granule cell layer (Figure 10). In contrast, the protein was more widespread, with very dark and uniform immunoreactivity in the molecular layer. The granule cell layer presented VGLUT1-IR punctate labeling corresponding to mossy fiber terminals. Finally, white matter contained faintly stained cell bodies reminiscent of interstitial cells. Although VGLUT2 mRNA was not detected in the cerebellum, protein was found throughout this region, but with a distribution that differed markedly from that of VGLUT1. The granule cell layer displayed the strongest signal, with VGLUT2-IR confined to mossy fiber terminals. The fainter immunoreactivity in the molecular layer was limited to sparse varicose fibers. As for VGLUT3 neither its transcript nor the protein were detected in the cerebellum. None of the VGLUTs (mRNA or protein) were expressed in the Purkinje cells.

## Discussion

The anatomical distribution of VGLUTs has been thoroughly described in rodents (see for example, Ni et al., 1995; Bellocchio et al., 1998, 2000; Aihara et al., 2000; Fremeau et al., 2001, 2002, 2004a,b; Gras et al., 2002, 2005, 2008; Kaneko and Fujiyama, 2002; Varoqui et al., 2002; Herzog et al., 2004; Hioki et al., 2004). However, only a few studies have been conducted in the human brain. Some of these studies analyzed the distribution of mRNA coding for VGLUTs (such as McCullumsmith and Meador-Woodruff, 2003; Oni-Orisan et al., 2008; Uezato et al., 2009; Van Der Hel et al., 2009); or investigated the presence of the proteins with subtype specific antisera (such as Kirvell et al., 2006; Kashani et al., 2007, 2008; Oni-Orisan et al., 2008; Van Der Hel et al., 2009) in a few brain regions. The current study aimed at localizing mRNA and protein of all three VGLUTs in various regions of the human brain, using respectively *in situ* hybridization and immunoautoradiography with VGLUT subtype-specific antisera. For this purpose, we developed the first human anti-VGLUT3 antiserum. This new anti-VGLUT3 provided the exact same immunolabeling pattern in rodent brain as that of a previously validated anti-mouse VGLUT3 antibody (Gras et al., 2008; Amilhon et al., 2010; Miot et al., 2012). A further indication of this serum's specificity was that it immunolabeled identical brain regions in rodents and humans, as discussed below. Furthermore, no labeling was observed when this antiserum was used on sections from mice no longer expressing VGLUT3 (Gras et al., 2008; Amilhon et al., 2010). These results strongly suggest that the newly developed antisera specifically recognize VGLUT3 and not the closely related VGLUT1 or VGLUT2 subtypes.

Our results are in line and extend previously published results. We found that transcripts coding for VGLUT1 and VGLUT2 were easily visualized. Interestingly, VGLUT1 and VGLUT2 transcripts are absent from area where both proteins are abundantly expressed (such as the caudate-putamen for example). This mismatch has been previously reported in rodent (Herzog et al., 2001) and reflects the fact that vesicular glutamate transporters are targeted to presynaptic terminals of principal neurons.

In human brains, VGLUT3 mRNA was hardly detected in some areas given weak signals and the small numbers of cells synthesizing this transcript. In contrast to VGLUT1 and VGLUT2, VGLUT3 transcript is often colocalized with the protein (in the hippocampus or caudate-putamen for example). This result suggests that, VGLUT3 has a discrete distribution in the human brain, similar to that previously described in rodents (Gras et al., 2002; Herzog et al., 2004).

As in rodents (Herzog et al., 2004), VGLUT3 mRNA was absent from the thalamus, the substantia nigra and the cerebellum whereas it was detected in sparse cells in the cortex, hippocampus, caudate-putamen, and in a more concentrated cell population within the raphe. The distribution of VGLUT3 transcript strongly suggests a localization in GABAergic interneurons in cerebral cortex and hippocampus, and in cholinergic interneurons in the caudate-putamen. This indicates a highly conserved expression pattern between rodents and humans. In the raphe, the VGLUT3-positive cell population appeared to be intermingled with 5-HT neurons but not totally overlapping the distribution of SERT mRNA. Here again suggesting that, as in rodents, VGLUT3 is expressed in a subpopulation of 5-HT neurons (Kiyasova et al., 2011). However, double-labeling experiments will be required to fully confirm these hypotheses. In contrast to the mRNA, VGLUT3-IR signal was found throughout gray mater areas with the exception of the cerebellum and thalamic nuclei. Interestingly, in the cerebral cortex, VGLUT3 seems to be more abundantly present in layer I–III and V–VI. This immunolabeling pattern is also highly similar to the one reported in rodent brain (Herzog et al., 2004; Somogyi et al., 2004). This implies that VGLUT3 is likely involved in the regulation of locomotor activity, reward and mood in humans, as previously suggested by studies conducted on mutant mouse models (Seal and Edwards, 2006; Gras et al., 2008; Amilhon et al., 2010). Since VGLUT3 is very discretely distributed in the human brain, it is a likely candidate for targeted treatments of specific pathologies such as Parkinson's disease, addiction, or mood disorders.

VGLUT1 and VGLUT2 patterns of mRNA expression in human brains corresponded to prominent cortical and subcortical glutamatergic systems, as previously described in rodents. In cerebral cortex, VGLUT1 mRNA displayed the most intense VGLUT signal. It was concentrated in layers V-VI, and thus presumably associated with pyramidal neurons projecting to subcortical structures. VGLUT2 mRNA, however, was rather weakly expressed in cortex, with signal being limited to a small band of cells in the middle layers. In contrast, protein expression for both transporter subtypes was strong in cerebral cortex. VGLUT1-IR yielded the strongest and most uniform staining in neocortex, spanning all layers homogeneously. VGLUT2-IR was also found throughout the cortical thickness, but much more weakly, except for a dense IR band in mid-cortex that colocalized with its mRNA distribution. This band also likely corresponded to layer V pyramidal neurons. The combined intensities of VGLUT1- and VGLUT2-IR throughout the cortical thickness clearly reflects the abundance of cortical and subcortical glutamatergic axon terminating in this structure. In the hippocampus, VGLUT1 (but not VGLUT2) was also associated with projection neurons. VGLUT1 mRNA sharply outlined the CA fields as well as the dentate gyrus, with strong signal in pyramidal neurons as well as granule cells. Thus, as in rodents, VGLUT1- and VGLUT2-IR suggests that VGLUT1 is the major vesicular glutamate transporter in the hippocampus and that VGLUT2 has only a discrete distribution in this area.

In subcortical regions, VGLUT1 and VGLUT2 displayed variable expression patterns. In the basal ganglia, although mRNAs for these transporters were completely absent, VGLUT1- and VGLUT2-IR were very intense in the caudate, putamen as well as the nucleus accumbens, and moderate in the substantia nigra. As previously described in rodents, VGLUT2-IR in the globus pallidus was also of moderate intensity, whereas VGLUT1 was absent. In the amygdala, the basolateral amygdaloid complex was the only region strongly delineated by VGLUT1- and VGLUT2-IR, as well as by VGLUT1 mRNA expression. Of note was the particularly high VGLUT2 mRNA and protein expression in the habenular complex, and moderate VGLUT1-IR in the lateral habenula. The only VGLUT mRNA (weakly) expressed in the thalamus was that coding for VGLUT2, with proportionally moderate immunoreactivity. The latter was uneven at the anatomical level examined, with sharp differences between thalamic nuclei. Heterogeneous VGLUT1-IR was also observed between nuclei, but its distribution did not match that of VGLUT2-IR.

The only VGLUT mRNA detected in the cerebellum was VGLUT1 mRNA strongly expressed in but confined to the granule cell layer. VGLUT1 and VGLUT2 proteins, however, were abundantly expressed and presented a complementary distribution along cerebellar layers. This strong IR is thus mainly provided by cerebellar afferents, among which the pontine nucleus was found to express high levels of VGLUT1 and VGLUT2 mRNA.

Similar to rodents (Herzog et al., 2004), VGLUT3 mRNA was absent from the thalamus, the substantia nigra and the cerebellum whereas it was detected in sparse cells in the cortex, hippocampus, caudate-putamen, and in a more concentrated cell population within the raphe. The distribution of VGLUT3 transcript strongly suggests a localization in GABAergic interneurons in cerebral cortex and hippocampus, and in cholinergic interneurons in the caudate-putamen. Again, this indicates a highly conserved expression pattern between rodents and humans. In the raphe, the VGLUT3-positive cell population appeared to be intermingled with 5-HT neurons but not totally overlapping the distribution of SERT mRNA. Here again suggesting that, as in rodents, VGLUT3 is expressed in a subpopulation of 5-HT neurons. However, double-labeling experiments will be required to fully confirm this hypothesis. In contrast to the mRNA, VGLUT3-IR signal was found throughout gray mater areas with the exception of the cerebellum and thalamic nuclei. Interestingly, in the cerebral cortex, VGLUT3 seems to be more abundantly present in layer I–III and V–VI. This immunolabeling pattern is also highly similar to the one reported in rodent brain (Herzog et al., 2004).

In summary, the anatomical distributions of brain cells expressing each one of the 3 VGLUTs are strikingly different. In contrast, the proteins are often found within the same areas (with the exception of the cererbellum and the thalamus, in which VGLUT3 was not detected). The regional distributions of VGLUT transcripts and proteins was found to be highly similar between human and rodent, suggesting that these transporters play fundamental roles in brain function that were conserved with evolution. This knowledge is particularly important given the previous implication of VGLUTs in cerebral pathologies, as it validates the use of rodent models to uncover the molecular mechanisms underlying human mental illnesses.

### Conflict of interest statement

The authors declare that the research was conducted in the absence of any commercial or financial relationships that could be construed as a potential conflict of interest.

## References

[B1] Aihara Y. Mashima H. Onda H. Hisano S. Kasuya H. Hori T.. (2000). Molecular cloning of a novel brain-type Na^(+)^-dependent inorganic phosphate cotransporter. J. Neurochem. 74, 2622–2625. 10.1046/j.1471-4159.2000.0742622.x 10820226

[B2] Amilhon B. Lepicard E. Renoir T. Mongeau R. Popa D. Poirel O.. (2010). VGLUT3 (vesicular glutamate transporter type 3) contribution to the regulation of serotonergic transmission and anxiety. J. Neurosci. 30, 2198–2210. 10.1523/JNEUROSCI.5196-09.2010 20147547 PMC6634029

[B3] Bellocchio E. E. Hu H. Pohorille A. Chan J. Pickel V. M. Edwards R. H. (1998). The localization of the brain-specific inorganic phosphate transporter suggests a specific presynaptic role in glutamatergic transmission. J. Neurosci. 18, 8648–8659. 9786972 10.1523/JNEUROSCI.18-21-08648.1998 PMC6793563

[B4] Bellocchio E. E. Reimer R. J. Fremeau R. T. Jr. Edwards R. H. (2000). Uptake of glutamate into synaptic vesicles by an inorganic phosphate transporter. Science 289, 957–960. 10.1126/science.289.5481.957 10938000

[B5] Benarroch E. E. (2010). Glutamate transporters: diversity, function, and involvement in neurologic disease. Neurology 74, 259–264. 10.1212/WNL.0b013e3181cc89e3 20083803

[B6] Boulland J. L. Jenstad M. Boekel A. J. Wouterlood F. G. Edwards R. H. Storm-Mathisen J.. (2009). Vesicular glutamate and GABA transporters sort to distinct sets of vesicles in a population of presynaptic terminals. Cereb. Cortex 19, 241–248. 10.1093/cercor/bhn077 18502731 PMC3202896

[B7] Daniels R. W. Collins C. A. Gelfand M. V. Dant J. Brooks E. S. Krantz D. E.. (2004). Increased expression of the Drosophila vesicular glutamate transporter leads to excess glutamate release and a compensatory decrease in quantal content. J. Neurosci. 24, 10466–10474. 10.1523/JNEUROSCI.3001-04.2004 15548661 PMC6730318

[B8] Eastwood S. L. Harrison P. J. (2010). Markers of glutamate synaptic transmission and plasticity are increased in the anterior cingulate cortex in bipolar disorder. Biol. Psychiatry 67, 1010–1016. 10.1016/j.biopsych.2009.12.004 20079890 PMC2868790

[B9] El Mestikawy S. Wallen-Mackenzie A. Fortin G. M. Descarries L. Trudeau L. E. (2011). From glutamate co-release to vesicular synergy: vesicular glutamate transporters. Nat. Rev. Neurosci. 12, 204–216. 10.1038/nrn2969 21415847

[B10] Fattorini G. Verderio C. Melone M. Giovedi S. Benfenati F. Matteoli M.. (2009). VGLUT1 and VGAT are sorted to the same population of synaptic vesicles in subsets of cortical axon terminals. J. Neurochem. 110, 1538–1546. 10.1111/j.1471-4159.2009.06251.x 19627441

[B11] Fonnum F. (1984). Glutamate: a neurotransmitter in mammalian brain. J. Neurochem. 42, 1–11. 10.1111/j.1471-4159.1984.tb09689.x 6139418

[B12] Fonnum F. Fykse E. M. Roseth S. (1998). Uptake of glutamate into synaptic vesicles. Prog. Brain Res. 116, 87–101. 10.1016/S0079-6123(08)60432-X 9932372

[B13] Fremeau R. T. Jr. Burman J. Qureshi T. Tran C. H. Proctor J. Johnson J.. (2002). The identification of vesicular glutamate transporter 3 suggests novel modes of signaling by glutamate. Proc. Natl. Acad. Sci. U.S.A. 99, 14488–14493. 10.1073/pnas.222546799 12388773 PMC137910

[B14] Fremeau R. T. Jr. Kam K. Qureshi T. Johnson J. Copenhagen D. R. Storm-Mathisen J.. (2004a). Vesicular glutamate transporters 1 and 2 target to functionally distinct synaptic release sites. Science 304, 1815–1819. 10.1126/science.1097468 15118123

[B15] Fremeau R. T. Jr. Troyer M. D. Pahner I. Nygaard G. O. Tran C. H. Reimer R. J.. (2001). The expression of vesicular glutamate transporters defines two classes of excitatory synapse. Neuron 31, 247–260. 10.1016/S0896-6273(01)00344-0 11502256

[B16] Fremeau R. T. Jr. Voglmaier S. Seal R. P. Edwards R. H. (2004b). VGLUTs define subsets of excitatory neurons and suggest novel roles for glutamate. Trends Neurosci. 27, 98–103. 10.1016/j.tins.2003.11.005 15102489

[B17] Gasnier B. (2000). The loading of neurotransmitters into synaptic vesicles. Biochimie 82, 327–337. 10.1016/S0300-9084(00)00221-2 10865121

[B18] Gras C. Amilhon B. Lepicard E. M. Poirel O. Vinatier J. Herbin M.. (2008). The vesicular glutamate transporter VGLUT3 synergizes striatal acetylcholine tone. Nat. Neurosci. 11, 292–300. 10.1038/nn2052 18278042

[B19] Gras C. Herzog E. Bellenchi G. C. Bernard V. Ravassard P. Pohl M.. (2002). A third vesicular glutamate transporter expressed by cholinergic and serotoninergic neurons. J. Neurosci. 22, 5442–5451. 12097496 10.1523/JNEUROSCI.22-13-05442.2002 PMC6758212

[B20] Gras C. Vinatier J. Amilhon B. Guerci A. Christov C. Ravassard P.. (2005). Developmentally regulated expression of VGLUT3 during early post-natal life. Neuropharmacology 49, 901–911. 10.1016/j.neuropharm.2005.07.023 16182324

[B21] Herzog E. Bellenchi G. C. Gras C. Bernard V. Ravassard P. Bedet C.. (2001). The existence of a second vesicular glutamate transporter specifies subpopulations of glutamatergic neurons. J. Neurosci. 21, RC181. 11698619 10.1523/JNEUROSCI.21-22-j0001.2001 PMC6762292

[B22] Herzog E. Gilchrist J. Gras C. Muzerelle A. Ravassard P. Giros B.. (2004). Localization of VGLUT3, the vesicular glutamate transporter type 3, in the rat brain. Neuroscience 123, 983–1002. 10.1016/j.neuroscience.2003.10.039 14751290

[B23] Hioki H. Fujiyama F. Nakamura K. Wu S. X. Matsuda W. Kaneko T. (2004). Chemically specific circuit composed of vesicular glutamate transporter 3- and preprotachykinin B-producing interneurons in the rat neocortex. Cereb. Cortex 14, 1266–1275. 10.1093/cercor/bhh088 15142960

[B24] Hnasko T. S. Edwards R. H. (2012). Neurotransmitter corelease: mechanism and physiological role. Annu. Rev. Physiol. 74, 225–243. 10.1146/annurev-physiol-020911-153315 22054239 PMC4090038

[B25] Kaneko T. Fujiyama F. (2002). Complementary distribution of vesicular glutamate transporters in the central nervous system. Neurosci. Res. 42, 243–250. 10.1016/S0168-0102(02)00009-3 11985876

[B26] Kashani A. Betancur C. Giros B. Hirsch E. El Mestikawy S. (2007). Altered expression of vesicular glutamate transporters VGLUT1 and VGLUT2 in Parkinson disease. Neurobiol. Aging 28, 568–578. 10.1016/j.neurobiolaging.2006.02.010 16563567 PMC1976623

[B27] Kashani A. Lepicard E. Poirel O. Videau C. David J. P. Fallet-Bianco C.. (2008). Loss of VGLUT1 and VGLUT2 in the prefrontal cortex is correlated with cognitive decline in Alzheimer disease. Neurobiol. Aging 29, 1619–1630. 10.1016/j.neurobiolaging.2007.04.010 17531353

[B28] Kirvell S. L. Esiri M. Francis P. T. (2006). Down-regulation of vesicular glutamate transporters precedes cell loss and pathology in Alzheimer's disease. J. Neurochem. 98, 939–950. 10.1111/j.1471-4159.2006.03935.x 16893425

[B29] Kiyasova V. Fernandez S. P. Laine J. Stankovski L. Muzerelle A. Doly S.. (2011). A genetically defined morphologically and functionally unique subset of 5-HT neurons in the mouse raphe nuclei. J. Neurosci. 31, 2756–2768. 10.1523/JNEUROSCI.4080-10.2011 21414898 PMC6623784

[B30] McCullumsmith R. E. Meador-Woodruff J. H. (2003). Expression of transcripts for the vesicular glutamate transporters in the human medial temporal lobe. Ann. N.Y. Acad. Sci. 1003, 438–442. 10.1196/annals.1300.046 14684482

[B31] Miot S. Voituron N. Sterlin A. Vigneault E. Morel L. Matrot B.. (2012). The vesicular glutamate transporter VGLUT3 contributes to protection against neonatal hypoxic stress. J. Physiol. 590, 5183–5198. 10.1113/jphysiol.2012.230722 22890712 PMC3497571

[B32] Moechars D. Weston M. C. Leo S. Callaerts-Vegh Z. Goris I. Daneels G.. (2006). Vesicular glutamate transporter VGLUT2 expression levels control quantal size and neuropathic pain. J. Neurosci. 26, 12055–12066. 10.1523/JNEUROSCI.2556-06.2006 17108179 PMC6674853

[B33] Ni B. Rosteck P. R. Jr. Nadi N. S. Paul S. M. (1994). Cloning and expression of a cDNA encoding a brain-specific Na^(+)^-dependent inorganic phosphate cotransporter. Proc. Natl. Acad. Sci. U.S.A. 91, 5607–5611. 10.1073/pnas.91.12.5607 8202535 PMC44045

[B34] Ni B. Wu X. Yan G. M. Wang J. Paul S. M. (1995). Regional expression and cellular localization of the Na^(+)^-dependent inorganic phosphate cotransporter of rat brain. J. Neurosci. 15, 5789–5799. 7643219 10.1523/JNEUROSCI.15-08-05789.1995 PMC6577628

[B35] Oni-Orisan A. Kristiansen L. V. Haroutunian V. Meador-Woodruff J. H. McCullumsmith R. E. (2008). Altered vesicular glutamate transporter expression in the anterior cingulate cortex in schizophrenia. Biol. Psychiatry 63, 766–775. 10.1016/j.biopsych.2007.10.020 18155679 PMC2669959

[B36] Ren J. Qin C. Hu F. Tan J. Qiu L. Zhao S.. (2011). Habenula “cholinergic” neurons co-release glutamate and acetylcholine and activate postsynaptic neurons via distinct transmission modes. Neuron 69, 445–452. 10.1016/j.neuron.2010.12.038 21315256

[B37] Ruel J. Emery S. Nouvian R. Bersot T. Amilhon B. Van Rybroek J. M.. (2008). Impairment of SLC17A8 encoding vesicular glutamate transporter-3, VGLUT3, underlies nonsyndromic deafness DFNA25 and inner hair cell dysfunction in null mice. Am. J. Hum. Genet. 83, 278–292. 10.1016/j.ajhg.2008.07.008 18674745 PMC2495073

[B38] Schafer M. K. Varoqui H. Defamie N. Weihe E. Erickson J. D. (2002). Molecular cloning and functional identification of mouse vesicular glutamate transporter 3 and its expression in subsets of novel excitatory neurons. J. Biol. Chem. 277, 50734–50748. 10.1074/jbc.M206738200 12384506

[B39] Seal R. P. Akil O. Yi E. Weber C. M. Grant L. Yoo J.. (2008). Sensorineural deafness and seizures in mice lacking vesicular glutamate transporter 3. Neuron 57, 263–275. 10.1016/j.neuron.2007.11.032 18215623 PMC2293283

[B40] Seal R. P. Edwards R. H. (2006). Functional implications of neurotransmitter co-release: glutamate and GABA share the load. Curr. Opin. Pharmacol. 6, 114–119. 10.1016/j.coph.2005.12.001 16359920

[B41] Somogyi J. Baude A. Omori Y. Shimizu H. El Mestikawy S. Fukaya M.. (2004). GABAergic basket cells expressing cholecystokinin contain vesicular glutamate transporter type 3 (VGLUT3) in their synaptic terminals in hippocampus and isocortex of the rat. Eur. J. Neurosci. 19, 552–569. 10.1111/j.0953-816X.2003.03091.x 14984406

[B42] Takamori S. Malherbe P. Broger C. Jahn R. (2002). Molecular cloning and functional characterization of human vesicular glutamate transporter 3. EMBO Rep. 3, 798–803. 10.1093/embo-reports/kvf159 12151341 PMC1084213

[B43] Takamori S. Rhee J. S. Rosenmund C. Jahn R. (2000). Identification of a vesicular glutamate transporter that defines a glutamatergic phenotype in neurons. Nature 407, 189–194. 10.1038/35025070 11001057

[B44] Takamori S. Rhee J. S. Rosenmund C. Jahn R. (2001). Identification of differentiation-associated brain-specific phosphate transporter as a second vesicular glutamate transporter (VGLUT2). J. Neurosci. 21, RC182. 11698620 10.1523/JNEUROSCI.21-22-j0002.2001 PMC6762262

[B45] Torres-Platas S. G. Cruceanu C. Chen G. G. Turecki G. Mechawar N. (2014). Evidence for increased microglial priming and macrophage recruitment in the dorsal anterior cingulate white matter of depressed suicides. Brain Behav. Immun. 42, 50–59. 10.1016/j.bbi.2014.05.007 24858659

[B46] Uezato A. Meador-Woodruff J. H. McCullumsmith R. E. (2009). Vesicular glutamate transporter mRNA expression in the medial temporal lobe in major depressive disorder, bipolar disorder, and schizophrenia. Bipolar Disord. 11, 711–725. 10.1111/j.1399-5618.2009.00752.x 19839996

[B47] Van Der Hel W. S. Verlinde S. A. Meijer D. H. De Wit M. Rensen M. G. Van Gassen K. L.. (2009). Hippocampal distribution of vesicular glutamate transporter 1 in patients with temporal lobe epilepsy. Epilepsia 50, 1717–1728. 10.1111/j.1528-1167.2009.02054.x 19389151

[B48] Varoqui H. Schafer M. K. Zhu H. Weihe E. Erickson J. D. (2002). Identification of the differentiation-associated Na^+^/PI transporter as a novel vesicular glutamate transporter expressed in a distinct set of glutamatergic synapses. J. Neurosci. 22, 142–155. 11756497 10.1523/JNEUROSCI.22-01-00142.2002 PMC6757588

[B49] Wallen-Mackenzie A. Gezelius H. Thoby-Brisson M. Nygard A. Enjin A. Fujiyama F.. (2006). Vesicular glutamate transporter 2 is required for central respiratory rhythm generation but not for locomotor central pattern generation. J. Neurosci. 26, 12294–12307. 10.1523/JNEUROSCI.3855-06.2006 17122055 PMC6675433

[B50] Wallen-Mackenzie A. Wootz H. Englund H. (2010). Genetic inactivation of the vesicular glutamate transporter 2 (VGLUT2) in the mouse: what have we learnt about functional glutamatergic neurotransmission? Ups. J. Med. Sci. 115, 11–20. 10.3109/03009730903572073 20187846 PMC2853350

[B51] Wojcik S. M. Rhee J. S. Herzog E. Sigler A. Jahn R. Takamori S.. (2004). An essential role for vesicular glutamate transporter 1 (VGLUT1) in postnatal development and control of quantal size. Proc. Natl. Acad. Sci. U.S.A. 101, 7158–7163. 10.1073/pnas.0401764101 15103023 PMC406482

[B52] Zander J. F. Munster-Wandowski A. Brunk I. Pahner I. Gomez-Lira G. Heinemann U.. (2010). Synaptic and vesicular coexistence of VGLUT and VGAT in selected excitatory and inhibitory synapses. J. Neurosci. 30, 7634–7645. 10.1523/JNEUROSCI.0141-10.2010 20519538 PMC6632366

